# Emergency Care for Refugee Patients at Suceava Hospital, Romania: Challenges and Insights from the First Year of the Russian-Ukrainian Conflict

**DOI:** 10.3390/healthcare13020138

**Published:** 2025-01-13

**Authors:** Elena Tătăranu, Laura Ion, Alexandru Nemțoi, Florin Filip, Sorin Axinte, Roxana Axinte, Monica Terteliu, Liliana Anchidin-Norocel, Smaranda Diaconescu

**Affiliations:** 1 “Sf. Ioan cel Nou” Clinical Emergency Hospital, 720237 Suceava, Romania; elena.tataranu@usm.ro (E.T.); florin.filip@usm.ro (F.F.); axintesorin@gmail.com (S.A.); roxaxinte@gmail.com (R.A.); monica.terteliu@usm.ro (M.T.); 2 Faculty of Medicine and Biological Sciences, Stefan cel Mare University of Suceava, 720229 Suceava, Romania; alexandru.nemtoi@usm.ro; 3 Faculty of Medicine, “Titu Maiorescu” University of Medicine, 031593 Bucharest, Romania; laura.ion@prof.utm.ro (L.I.); smaranda.diaconescu@prof.utm.ro (S.D.)

**Keywords:** migrants, health care, children

## Abstract

Background: The ongoing military conflict in Ukraine has had a devastating impact on children’s health, exposing them to a range of illnesses. The aim of this study was to analyze the most common medical conditions among Ukrainian children since the beginning of the conflict, with a focus on identifying and understanding these problems in a wartime setting. Method: To assess the health status of affected children, we collected data from 422 pediatric patients who presented to the emergency department. The analysis included reviewing medical records, documenting the nature of illness, treatments administered, and the need for hospitalization. Results: Preliminary results indicate that interstitial pneumonia, contusions, gastroenterocolitis, and traumatic brain injury were the most common conditions. Of the 422 children studied, 80% received appropriate care without hospitalization, while 20% were admitted for further evaluation. Conclusions: Interstitial pneumonia was diagnosed in 23% of patients, highlighting the vulnerability of the respiratory system under conflict conditions. Contusions were predominant among musculoskeletal injuries, accounting for 81% of cases, and gastroenterocolitis was diagnosed in 46% of patients, reflecting the impact of poor living conditions.

## 1. Introduction

**Figure 1:**
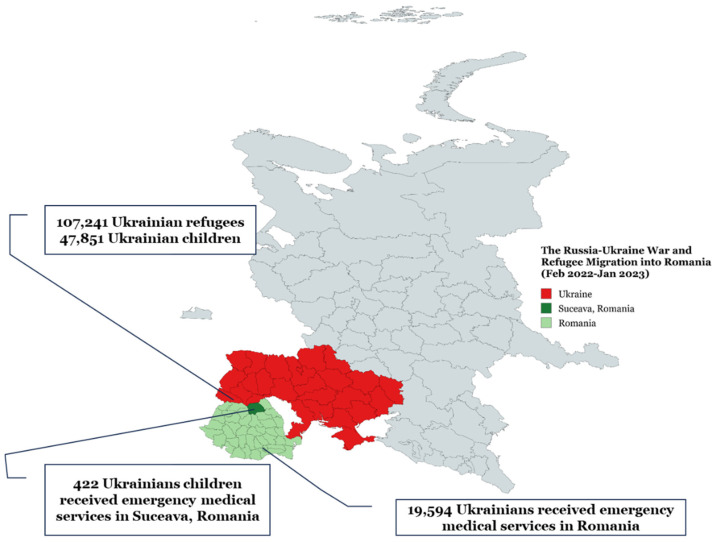
Refugee migration to Romania (2022–2023) and demand for emergency medical services (generated with https://www.mapchart.net/index.html, accessed on 13 November 2024).

On 24 February 2022, Russia escalated its military operations in Ukraine by launching a large-scale offensive across the country [1]. The war has not only resulted in immediate casualties but has also significantly disrupted Ukraine’s public health system and hospital infrastructure [2]. The prolonged nature of the conflict has exacerbated shortages in medical supplies, placed immense pressure on healthcare workers, and triggered public health emergencies, while contributing to a severe mental health crisis among the Ukrainian population [3]. The Ukraine war has triggered an enormous humanitarian crisis that has inflicted, and continues to inflict, deep and enduring harms on human health [4]. The pediatric population represents one of, if not the most, vulnerable groups affected by the Ukrainian crisis [5]. There are physical risks for children that live in war zones, such as breathing in smoke and ash from fires and blasts that can affect the nose and lungs [6]. Romania began receiving refugees on 24 February 2022. Since then, until 2024, over 1.6 million Ukrainians have entered the country, with approximately 83,748 still residing in Romania. About 74,000 of these refugees have been granted temporary protection. Recently, there has been an increasing trend of Ukrainians crossing back into their home country, with the total number returning to Ukraine reaching approximately 1,091,000 to date. Since the onset of the war, a Ukrainian child has become a refugee nearly every second [7]. It is estimated that around 33% of Ukrainian refugees in Romania are children. In some instances, children have been separated from their parents or guardians before their journey to Romania, traveling in groups with distant relatives or strangers, as their family members returned to Ukraine after dropping them off. Since February 24, approximately 4620 unaccompanied children have arrived in Romania, with 202 currently in the state protection system [8]. Refugee migration to Romania (2022–2023) and the demand for emergency medical services are presented in Figure 1.

Prior to Emergency Ordinance no. 20/2022 of Romania on humanitarian assistance for those fleeing Ukraine, Ukrainian citizens and other beneficiaries of temporary protection in Romania did not have access to specific social security benefits. However, they were entitled to emergency medical care. With the introduction of GEO 20/2022, the provisions for assistance and medical care were updated to include exceptions for individuals arriving from the conflict zone in Ukraine. These individuals are now eligible for the basic package of services under the social health insurance system, similar to insured Romanian citizens, without the obligation to pay social health insurance contributions or co-payments [9,10]. The “Sfântul Ioan cel Nou” Clinical Emergency County Hospital of Suceava is the most important healthcare facility in the county, with a capacity of 1200 beds and 31 wards, offering comprehensive, high-quality medical services across most pathologies treated at the national level. It plays a critical role in coordinating emergency medical assistance in collaboration with the County Inspectorate for Emergency Situations. During the COVID-19 pandemic, it was one of the first hospitals in Romania to be significantly affected, highlighting its critical role in managing healthcare crises.

## 2. Health Impacts of Crises on Refugee Populations

### 2.1. Physical Health Challenges in Refugee Children

It is known that the general health of refugee children is worse than that of non-immigrant children. Among the most serious health problems are considered to be infectious diseases (tuberculosis, measles, malaria, hepatitis B, HIV, and intestinal parasites), but also other diseases presented in Table 1 [11].

### 2.2. Mental Health Burdens in Conflict Zones

Armed conflict has a devastating impact on the mental health of affected populations. Post-traumatic stress disorder and depression are the most common post-war mental disorders in adults and children [21,22]. Up to one-third of people who have directly experienced the trauma of war develop these conditions. Exposure to different types of violence, the duration of the conflict, and the types of traumatic events experienced and observed are all associated with the onset and severity of psychological disorders in children affected by conflict [23]. Identified vulnerabilities are often interrelated and can have an impact on children (direct and indirect exposure, cognitive abilities), families (loss of loved ones, prolonged separation, parental mental health, and unemployment), and communities (lack of basic needs such as food and water, disruption or damage to support networks, persistent poverty, discrimination) [24].

### 2.3. Healthcare Systems Under Pressure

During times of war, healthcare systems operate under immense and sustained pressure, far beyond the challenges faced during peacetime. In addition to existing issues like aging populations, chronic health conditions, and the high standard of care expected by society, war introduces acute pressures such as mass casualties, destruction of healthcare infrastructure, and limited access to essential supplies [25].

**Figure 2:**
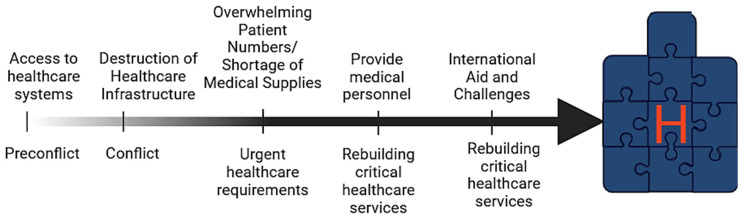
Stages of healthcare system under pressure during wartime.

The stages of the healthcare system under pressure during wartime (Figure 2) begin with access to healthcare systems in the preconflict stage, followed by the destruction of healthcare infrastructure, urgent healthcare needs, the provision of medical personnel, the rebuilding critical healthcare services, and addressing international aid and challenges. Healthcare institutions in war zones must balance overwhelming demand for care with limited resources. Emergency services are often overwhelmed by surges of patients, where treatment needs far outstrip available staff, equipment, or safe physical spaces [26]. Teams and individual professionals must constantly adapt, reallocating staff, adjusting patient flow, and reorganizing service delivery to respond to the evolving crisis. These adaptations aim to minimize harm, provide essential care, and maintain basic safety and quality of services under dire constraints [27].

An important opportunity to access the healthcare system in such war or crisis situations is telemedicine, which is promoted and supported by the governments of some affected countries. Telemedicine proves to be an invaluable tool in challenging and dangerous situations, such as conflict zones and natural disasters. It enables individuals in high-risk areas to access medical care remotely. In conflict zones, telemedicine is particularly beneficial for treating injured or ill soldiers and providing psychological support to help them manage the stress of combat [28].

The purpose of our study is to analyze and describe the medical care received by refugee children from Ukraine in emergency departments, since the first days after the outbreak of the conflict. We aim to identify the demographic characteristics of these children, evaluate the types of pathologies encountered, and examine how they are redirected to various medical specialties. This research enables emergency departments to respond more effectively to the challenges faced by this vulnerable population. In the same year as our study (2022–2023), there were 334 attacks on 267 Ukrainian health facilities; 230 facilities were damaged and 37 were destroyed. General hospitals, primary care clinics, emergency departments, and children’s hospitals were most commonly targeted. Most of the attacks took place in the first three months and in the regions of eastern Ukraine [10].

## 3. Materials and Methods

This study is a retrospective analysis focusing on the impact of the Ukraine conflict on pediatric pathology. It examined a cohort of 422 refugee pediatric patients (ages 0–18) who presented to the Emergency Unit at “Sfântul Ioan cel Nou” County Hospital in Suceava between February 2022 and January 2023. The inclusion criteria for the study were as follows: refugees who had previously resided in Ukraine, being within the analyzed time period, being of pediatric age, and having informed consent signed by their parents. The exclusion criteria consisted of failing to meet any of the aforementioned inclusion criteria. To facilitate communication with Ukrainian refugees, a translator was available in the Emergency Unit, ensuring that patients and their families could effectively convey their medical concerns and understand the care provided. The study period was selected based on the significant drop in patient numbers observed in 2023, as many refugees either relocated to other cities within Romania or left the country for other European Union destinations. This Emergency Unit typically manages over 20,000 pediatric cases annually. Since the Ukraine-Russia conflict began, the patient volume has grown, driven in part by the arrival of child refugees from Ukraine. The data were collected from the hospital’s storage program, Info-world, with a focus on identifying common pathologies among these patients, including respiratory conditions, cranio-cerebral traumas, digestive disorders, and musculoskeletal issues. This study was approved by the Ethical Committee of “Sf. Ioan cel Nou” County Hospital, Suceava, Romania, nr 61/28.11.2024. All patients provided informed consent upon presenting to the Emergency Unit, and for minors, this consent was completed by their parents or legal guardians, ensuring compliance with ethical standards.

## 4. Results

**Figure 3:**
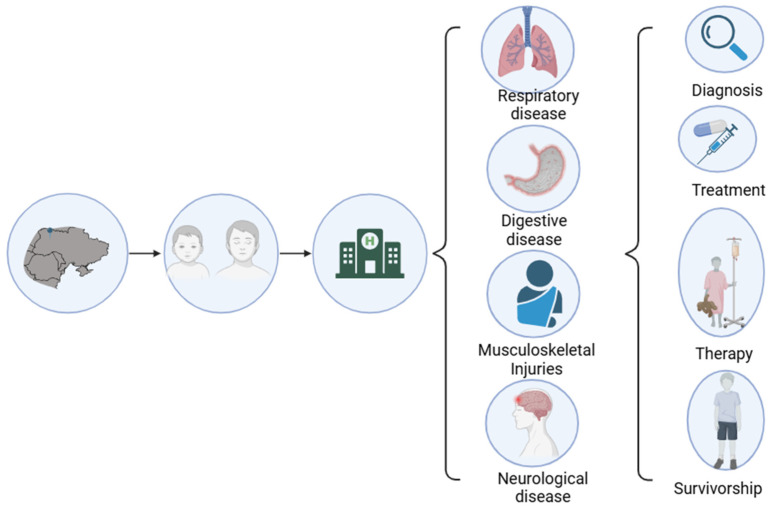
Ukrainian refugee children pathway of the Emergency Unit.

All pediatric patients who were refugees from Ukraine and presented to the Emergency Unit at “Sfântul Ioan cel Nou” County Hospital in Suceava underwent a comprehensive process of diagnosis, treatment, therapy, and survivorship care (Figure 3).

The data analysis (Table 2) highlights the distribution of refugee children by age group and the departments to which they were redirected at the Emergency Unit in Suceava. In the 0–3 years age group, out of a total of 138 patients, a significant majority of 133 children (96%) were directed to the Pediatric Holding Department. Additionally, two children (1%) were sent to the Infectious Diseases Department, with one child (1%) each referred to General Pediatrics, COVID Pediatrics, and Neurosurgery. These findings suggest that most young children required general pediatric care, with only a small proportion needing more specialized interventions.

**Figure 4:**
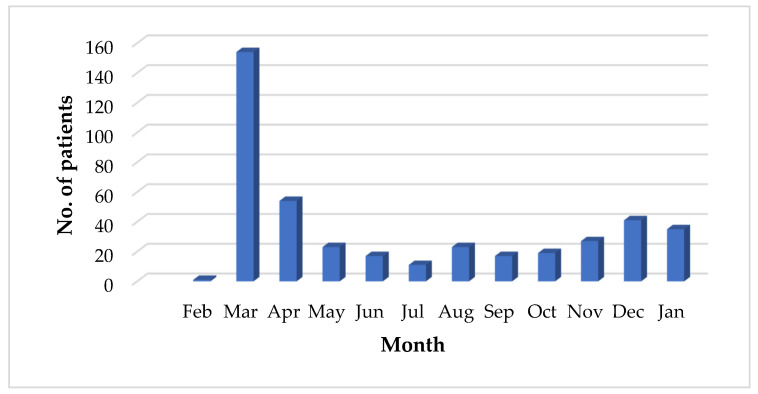
Frequencies of patients in emergency care according to month.

For children aged 4–7 years, out of the 126 patients evaluated at the Emergency Unit, 119 (94%) were redirected to the Pediatric Holding Department, indicating a predominant need for care that could be addressed within this unit. The remainder were directed to Pediatric Neurology (three children, 2%), General Pediatrics (two children, 2%), Infectious Diseases (one child, 1%), and Neurosurgery (one child, 1%). In the 8–14 years age group, among 113 patients, most (61 children, 54%) were admitted to the Pediatric Holding Department, while others were distributed across General Pediatrics (22%), Pediatric Surgery and Orthopedics (21%), and other specialties, suggesting an increasing variety of pathologies with age. For adolescents aged 15–18 years (45 patients), the distribution was more diverse: 42% were directed to Pediatric Surgery and Orthopedics, 27% to the Pediatric Holding Department, and 25% to Obstetrics-Gynecology, with isolated cases redirected to General Pediatrics, General Surgery, and Pediatric Neurology. These findings indicate a higher demand for surgical and gynecological specialties in adolescents compared to younger children, reflecting an increasing complexity of pathologies as age increases. Of the entire cohort, 20% were admitted to the hospital. Out of a total of 422, 80% of Ukrainian pediatric patients were seen in the Emergency Unit, received consultations, received appropriate medication for their conditions, and were discharged without the need for hospitalization. Meanwhile, only 20% of patients required admission for further investigations and additional tests. The most frequent pathologies of Ukrainian pediatric patients were respiratory diseases, present in 142 children, musculoskeletal injuries in 89 children, digestive diseases in 79 children, neurological diseases in 34 children, and 78 patients with other diseases. Figure 4 illustrates the monthly distribution of Ukrainian child patients treated at our hospital, highlighting a significant increase in cases during the first months following the onset of the conflict, followed by a gradual decline. Additionally, there is a noticeable rise in patient numbers as winter approaches, likely due to seasonal factors that contribute to increased healthcare needs.

**Figure 5:**
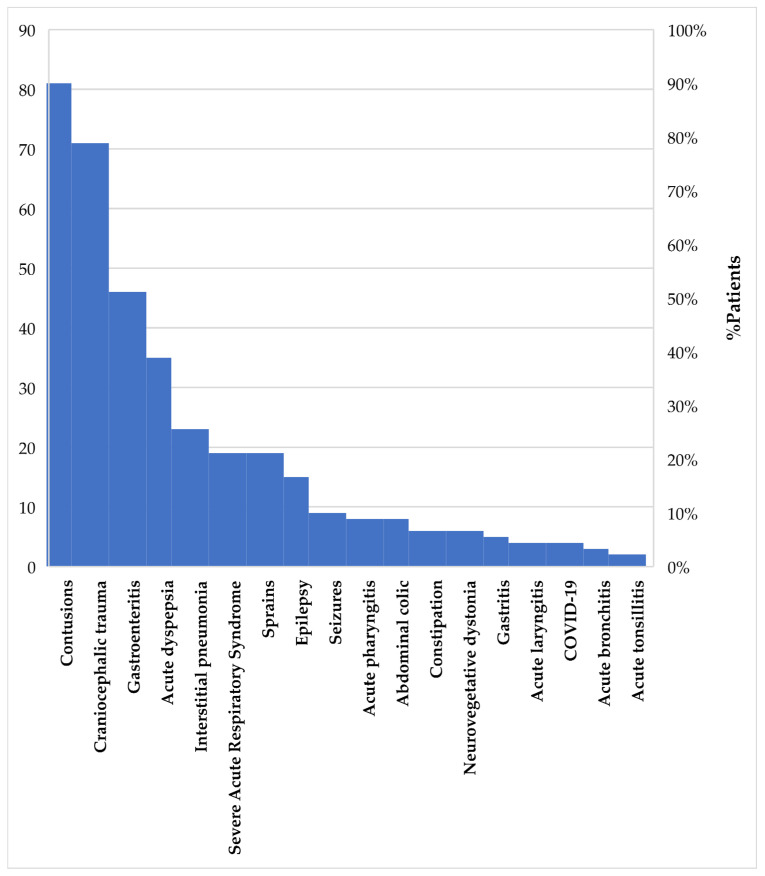
The most common diagnoses in respiratory, digestive, and neurological disorders, and musculoskeletal injuries.

According to the extracted data (Figure 5), interstitial pneumonia is the most common condition, accounting for 23% of respiratory disorders, followed by severe acute respiratory syndrome (SARS) at 19%. The graphical results regarding musculoskeletal injuries show that contusions represent 81%, while sprains account for 19%, and gastroenterocolitis emerges as the most frequent diagnosis among digestive disorders, with a prevalence of 46%, followed by acute dyspepsia at 35%. Regarding neurological disorders, out of a total of 34 pediatric patients, 23 suffered from traumatic brain injury, representing 71%. Five patients had epilepsy, accounting for 15%, while three had seizures (9%), and two patients experienced neurovegetative dystonia, making up 6%. The majority of patients included in the study were evaluated in the emergency department, so we do not have much data on associated comorbidities, but the records of hospitalized patients, specified that there were two cases of hydrocephalus with ventriculoperitoneal shunting, one case of common variable immunodeficiency, and five cases of cardiac malformations.

## 5. Discussion

The Government of Romania, together with its partners, provided medical services, supplies, medicines, and medical devices through the social health insurance system, ensuring the full integration of Ukrainian refugees into the healthcare system. By January 2023, out of a total of 107,241 Ukrainian refugees in the country, 47,851 were children. In Romania, 19,594 Ukrainians received emergency medical services, of whom 3170 were hospitalized, and in Suceava, there were 422 cases involving children [29].

The negative effects of armed conflicts on children’s health have been extensively documented and seem to affect children’s physical, mental, and behavioral health [30,31]. War inflicts suffering, trauma, illness, and collapse of organizations responsible for preventive, palliative, and curative care for the population, with its effects deeply reverberating across society [32]. The mediators of loss include the decline of healthcare services, educational setbacks, disruptions in supply, economic downturns, and the forced displacement of populations—all indirect effects with a pervasive impact that may extend to future generations [33].

From this, we can observe the implications of this war, particularly on children, who have required specialized care across various medical fields, as well as the impact on their emotional well-being, given that children are especially vulnerable to illness and stress caused by instability and insecurity. Regarding the most common conditions in the respiratory category, interstitial pneumonia was the most frequently encountered among children, accounting for 23%, in contrast to acute tonsillitis, which was less common at only 2%. It is known that, particularly in conflict zones, viral agents (such as Respiratory Syncytial Virus, Rubella, Adenovirus, Measles), refs. [34,35,36], bacterial agents (like Koch’s bacillus, Listeria, Chlamydia) [37], parasitic agents (Toxoplasma), as well as immune system dysfunctions can lead to hypersensitivity in the body. As a result, the lungs’ ability to inhale oxygen and the exchange of oxygen between systems are no longer functioning normally. Prolonged exposure to high levels of air pollution can hinder children’s lung function and increase the risk of respiratory infections. In the present study, we could not establish correlations between interstitial pneumonia and exposure to harmful substances, as we do not have precise information about the refugees’ places of origin. However, we can link the high prevalence of pneumonia to the fact that many of these children initially stayed in overcrowded conditions, such as refugee centers, particularly during the winter season.

Given the context of the ongoing war in Ukraine, locomotor conditions are particularly frequent due to intense fighting, explosions, and the fear and terror that compel people to flee from imminent danger. Analyzing the data, gastroenterocolitis is the most frequent diagnosis among digestive disorders, accounting for 46%, in contrast to gastritis, which only has a prevalence of 5%. Considering the difficult conditions during the war and public health deficiencies, gastroenterocolitis is commonly seen during military conflicts, as it represents a common infectious disease syndrome with a multitude of symptoms such as nausea, vomiting, diarrhea, and abdominal pain. It can be caused by viral agents (such as *Rotavirus*, *Norovirus*, *Astrovirus*) [38,39], bacterial agents (like *Salmonella*, *E. coli*, *Clostridium difficile*) [40], and parasites (including *Giardia*, *Cyclospora*, *Amoebiasis*) [41,42]. It is evident that due to the military conflict, people have been living in inhumane conditions, in overcrowded, poorly ventilated spaces with excessive humidity, consuming improperly stored food and contaminated water, which has led to the spread of viral, bacterial, and parasitic agents [43]. Consequently, this has resulted in an increase in gastroenterocolitis, as it can easily spread from person to person, especially when basic hygiene practices are not followed [44]. According to statistical data regarding neurological disorders, traumatic brain injuries (TBI) have been the most common, with a prevalence of 71%, compared to neurovegetative dystonia, which has only 6%. The war is the primary cause of this increased incidence of TBI, as the extreme violence and terrorism associated with this unjust military operation have directly and indirectly led to a surge in TBI cases, particularly among children who have become collateral victims. The direct implications of the war in Ukraine concerning traumatic brain injuries are marked by physical violence (beatings, terrorism, kidnappings) and sexual violence against children. Indirect implications (such as shootings, grenades, explosions) have also had repercussions, leading to injuries and traumatic brain injuries [45]. The study of Milkova et al., 2023, and our research focus on the medical care of refugee children in Ukraine, highlighting important similarities in the distribution by age groups and the typology of pathologies. Both studies show that the majority of patients fall into the 0–7 age group, which has the greatest need for pediatric care, with predominant respiratory conditions. Both studies also emphasize the importance of outpatient care, with most children requiring only consultations without hospitalization, reflecting a prevalence of mild to moderate cases [46]. The health issues identified by authors in the case of refugees globally are similar to those observed in our study, including respiratory infections, trauma, and gastrointestinal conditions.

## 6. Conclusions

The consequences of armed conflicts on public health, both short term and long term, are well documented, with several specific features of these conflicts being analyzed, including infections, malnutrition, violence in all its forms, and mental health challenges. The suffering caused by these conflicts permeates through multifaceted permissive environments, encompassing cultural, economic, and biological factors, as well as societal vulnerabilities and individual resilience, especially when hostilities are prolonged. Since the beginning of the military conflict, interstitial pneumonia, contusions, gastroenterocolitis, and traumatic brain injury have been the most common diseases among children in Ukraine. Of the children admitted to the emergency room, the majority were evaluated and treated without requiring hospitalization. A smaller portion of patients needed hospital admission for further diagnostic investigations and specialized treatment. Among respiratory diseases, interstitial pneumonia emerged as the most frequent condition, while acute tonsillitis was less commonly encountered. This is because in conflict areas, infectious agents can trigger allergies in the body, affect lung function, and increase the risk of respiratory infections, especially in the presence of pollution and overpopulation. Among musculoskeletal injuries, contusions were the most common, reflecting the impact of the violence and fear of the conflict on children. In addition, patients diagnosed with gastroenterocolitis highlight the prevalence of infectious diseases due to the poor living conditions and poor hygiene common during armed conflict. Finally, traumatic brain injury was found in a high percentage of the children studied, highlighting the extremely high risks children face during conflict, both from direct violence and secondary effects of war situations. These statistics reflect not only the urgent need for medical intervention, but also the long-term impact on the physical and mental health of children affected by the conflict in Ukraine.

## Figures and Tables

**Table 1:** Health concerns in refugee children.

Health Concerns	Number of Children Refugees	Year	Reference
Anemia and genetic disorders of the red blood cells Infectious diseases Growth and nutrition Vaccine coverage	Review		[12]
Tuberculosis, hepatitis B, and vaccine-preventable and parasitic diseases	Review		[13]
Anemia Burn injuries and other types of significant trauma	63 patients	2005–2015	[14]
Infectious diseases Gastrointestinal disorders Surgical disorders Allergic disorders	2631 patients Syrian	2016–2017	[15]
Post-traumatic stress (i.e., sexual violence, domestic violence)	1.5 million Syrian	Since 2012	[16]
Mental health problems	10 patients Afghan	2021–2022	[17]
Dental problems Post-traumatic stress and sleeping problems	639 patients Afghan or Syrian	2015	[18]
Respiratory and Infectious diseases Diseases of the skin, ear, or gastrointestinal tract	1423 patients	2015–2016	[19]
Malnutrition	176 patients	2017	[20]

**Table 2:** Characteristics of the patients (n = 422).

Age Distribution (Years)	n (%)
0–3 years	138 (32.70%)
4–7 years	126 (29.86%)
8–14	113 (26.78%)
15–18	45 (10.66%)
**Environment**	
urban	143 (34%)
rural	279 (66%)
**Reason to visit**	
respiratory diseases	142 (33.65%)
musculoskeletal injuries	89 (21.09%)
neurological diseases	34 (8.06%)
digestive diseases	79 (18.72%)
others (i.e., balanitis, ingrown toenail)	78 (18.48%)

## Data Availability

The data presented in this study are available on request from the corresponding author due to the large amount of data and privacy concerns.
